# An integrated study of human and animal infectious disease in the Lake Victoria crescent small-holder crop-livestock production system, Kenya

**DOI:** 10.1186/s12879-017-2559-6

**Published:** 2017-06-30

**Authors:** Eric M. Fèvre, William A. de Glanville, Lian F. Thomas, Elizabeth A. J. Cook, Samuel Kariuki, Claire N. Wamae

**Affiliations:** 1 Institute of Infection and Global Health, University of Liverpool, Leahurst Campus, Neston, CH64 7TE UK; 2 International Livestock Research Institute (ILRI), Old Naivasha Road, PO Box 30709-00100, Nairobi, Kenya; 3 Centre for Immunity, Infection and Evolution, Institute for Immunology and Infection Research, School of Biological Sciences, Kings Buildings, University of Edinburgh, West Mains Road, Edinburgh, EH9 3JT UK; 4 Centre for Microbiology Research, Kenya Medical Research Institute, PO Box 43640, Nairobi, Kenya; 5 Current address: Department of Microbiology, School of Medicine, Mount Kenya University, PO BOX 342-00100, Thika, Kenya

**Keywords:** Zoonoses, One health, *Taenia*, *Coxiella*, *Mycobacterium*, *Brucella*, Kenya

## Abstract

**Background:**

The neglected zoonotic diseases (NZD) are an understudied group that are a major cause of illness throughout the developing world. In general, little is known about the prevalence and burden of NZDs in affected communities, particularly in relation to other infectious diseases with which they are often co-endemic. We describe the design and descriptive epidemiological outputs from an integrated study of human and animal zoonotic and non-zoonotic disease in a rural farming community in western Kenya.

**Methods:**

This cross-sectional survey involved 2113 people, their cattle (*n* = 983) and pigs (*n* = 91). People and animals were tested for infection or exposure to a wide range of zoonotic and non-zoonotic pathogens. Prevalence estimates, with adjustment for the complex study design, were derived. Evidence for spatial clustering in exposure or infection was identified using the spatial scan statistic.

**Results:**

There was a high prevalence of human parasitism in the community, particularly with hookworm *(Ancylostoma duodenale* or *Necator americanus*) (36.3% (95% CI 32.8–39.9)), *Entamoeba histolytica/dispar* (30.1% (95% CI 27.5–32.8)), and *Plasmodium falciparum* (29.4% (95% CI 26.8–32.0)). Human infection with *Taenia* spp. was also prevalent (19.7% (95% CI 16.7–22.7)), while exposure to other zoonotic pathogens was comparatively rarer (*Brucella* spp., 0.6% (95% CI 0.2–0.9); *Coxiella burnetii,* 2.2% (95% CI 1.5–2.9); Rift Valley fever, 0.5% (95% CI 0.2–0.8)). A low prevalence of exposure to *Brucella* spp. was observed in cattle (0.26% (95% CI 0–0.56). This was higher for Rift Valley fever virus (1.4% (95% CI 0.5–2.22)) and *C. burnetii* (10.0% (95% CI 7.7–12.2)). The prevalence of *Taenia* spp. cysticercosis was 53.5% (95% CI 48.7–58.3) in cattle and 17.2% (95% CI 9.1–25.3) in pigs. *Mycobacterium bovis* infection was found in 2.2% of cattle (95% CI 1.3–3.2), while the prevalence of infection with *Mycobacterium* spp. was 8.2% (95% CI 6.8–9.6) in people.

**Conclusion:**

Zoonotic infections in people and animals occur in the context of a wide range of co-endemic pathogens in a rural community in western Kenya. The wide diversity of pathogens under study provides a unique opportunity to explore the distribution and determinants of infection in a multi-pathogen, multi-host system.

## Background

Zoonotic diseases are caused by a diverse group of pathogens that are transmissible from animals to humans. Several of these diseases, such as avian influenza and bovine spongiform encephalopathy, are extensively studied and are the focus of large scale and successful control efforts. Another group, the so-called neglected zoonotic diseases (NZDs), are under-researched and under-funded at several levels, and tend to be poorly understood [1]. For these diseases, key biological and epidemiological data on occurrence, burden and risk, in both animals and humans, are lacking, particularly in low and middle income countries. In addition, reliable, cheap and easy-to-use tools for diagnosis and control are either not available or are poorly applied [2].

The World Health Organization (WHO) has highlighted the inadequacy of the evidence base for decision making relating to zoonoses in resource poor settings [2]. Identified as being particularly important in addressing the information gap were: field epidemiological studies in humans and livestock; estimates of under-reporting; multi-disease studies in communities; development of field-level diagnostics; intervention cost-effectiveness studies; and improved understanding of pathogen and host ecology [2, 3].

We present here the outputs from a novel epidemiological investigation that sought to address some of these information gaps in the Lake Victoria Crescent zone of western Kenya. Disease-specific data on a number of neglected zoonotic infections were collected concurrently from domestic animals and people living in the same households. The “People, Animals and their Zoonoses” (PAZ) project is a collaborative venture between universities and research institutes in Europe and Kenya, and subscribes to the ‘One Health’ [4] framework of interdisciplinary research by considering disease in livestock and humans concurrently.

We selected a number of zoonotic diseases that were expected to cause a significant burden to livestock-keeping communities in the region [5]. These were brucellosis, Q-fever, bovine tuberculosis, human African trypanosomiasis (HAT), Rift Valley fever (RVF), and cysticercosis/taeniasis. Contact with livestock or their products is a risk factor for human infection with the aetiological agent of each disease, with positive associations reported for prevalence of infection [6–9], although the precise nature of the relationship is not always clear [10]. Many of the chosen zoonoses are also significantly under-reported in livestock and humans in endemic areas [11, 12]. As individual diseases, they are typically not priorities for medical or veterinary services, or indeed the research community [2], even if the diseases and their sequelae [13, 14] result in a high burden [15–17]. This study aimed to establish estimates of exposure to infection at the population level with a range of pathogens in a smallholder, mixed crop and livestock production system. As such, and unlike many previous studies, it does not focus on known at-risk groups (e.g. specifically on slaughterhouse workers or people attending hospital), but on capturing data from otherwise healthy people and their livestock at the household level.

Co-infection with zoonotic and other pathogens is likely to be a frequent occurrence in poor communities in tropical and sub-tropical Africa, imposing a combined but typically unquantified burden. Such communities may be coping with a wide range of endemic infectious diseases in both people (e.g. malaria, soil transmitted helminthiasis, schistosomiasis, tuberculosis, HIV) and their animals (e.g. East Coast fever (ECF), helminthiasis, trypanosomiasis, bovine tuberculosis). These ‘co-factors” may exacerbate susceptibility to zoonotic agents in individuals or result in enhanced spread at a community level [18]. To further explore this dual burden, we also quantified the prevalence of a wide range of endemic, non-zoonotic infectious agents in both people and animals within the study population. The PAZ project therefore represents a holistic, multi-pathogen, multi-host study of infectious disease within a single community that seeks to simultaneously understand zoonotic and other disease burdens, the distribution of infection, and determinants of infection in both people and their livestock.

Here we describe the design of this integrated study of human and animal health. We report on the descriptive epidemiology of infection with a range of endemic diseases in people and animals, as well as on the demographic characteristics of the population under study that may influence its zoonotic disease risk.

## Methods

### Study area

**Fig. 1:**
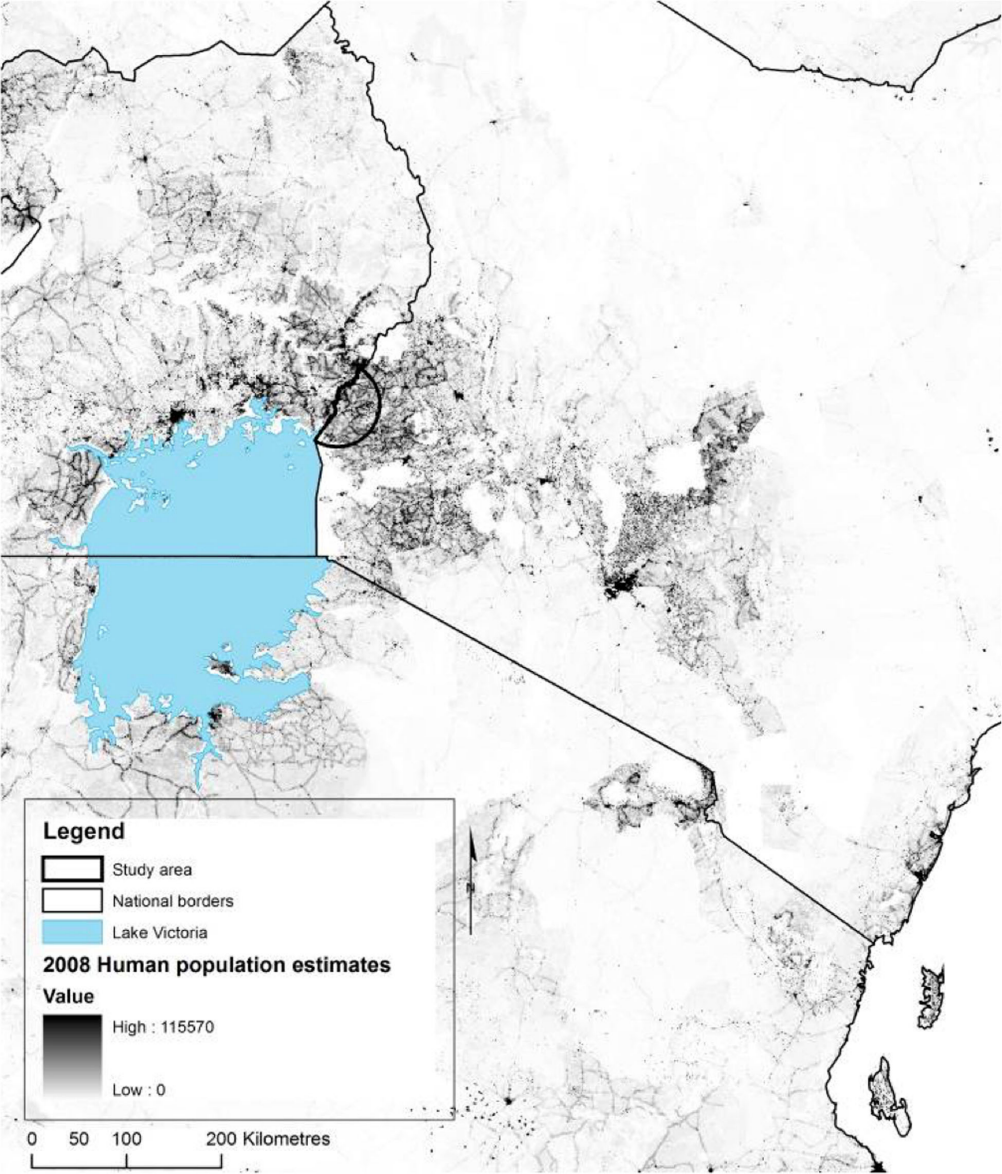
Study area shown in the context of human population density in eastern Africa [60]

The study population was a mixed-farming community in western Kenya in an area broadly representative of the wider Lake Victoria Crescent ecosystem which spans Kenya, Uganda and Tanzania (Fig. 1). This region is characterised by rainfall and temperatures that are typically sufficient for two cropping seasons per year, and in which a range of subsistence and cash-crops are grown by the majority of rural households. Livestock, specifically local breeds of cattle, sheep and goats, and smaller numbers of pigs, are integrated with crop production in a mixed farming system through the use of manure as fertiliser, cattle as draft power, and crop surplus and residues as animal feed [19].

The study area was an approximately 3200 km^2^ zone defined by a semi-circle with a 45 km radius emanating from the county town of Busia (in which the study field station was situated) on the Kenyan border with Uganda (Fig. 1). This area comprises a human population of 1.4 million people (OpenData, http://www.opendata.go.ke/) a cattle population of around 340,000 head and a domestic pig population of around 55,000 (Divisional Livestock Production Office data (DPLO)).

### Study design

The study was cross-sectional, in which the primary sampling unit was the household (locally called a homestead), within which all eligible and consenting members and their livestock were recruited. The study sample size was powered to estimate the prevalence of zoonotic disease in cattle, with the human sample incidental to that for cattle, but expected to be larger. We used a standard error of 2% for the lowest expected prevalence of a bovine infection or exposure of 5%, and a design effect of 3 to inflate sample size estimates to account for expected clustering at the household level. The resulting cattle sample size of 1365 head were expected to be found in a random sample of 412 households, based on local estimates of herd size and the frequency of cattle ownership. Field data collection took place continually between August 2010 and July 2012.

### Household selection

Random sampling of households was stratified by sub-location, the smallest administrative unit in Kenya. There were 143 sublocations in the study area at the time of the study, and the number of households to select per sublocation was proportional to the expected number of cattle in the sublocation, with a minimum of 1 and a maximum of 8. Sampling proportional to number of cattle in a sublocation ensured cattle (and cattle keeping households) had an equal probability of being selected across the whole study area. Cattle population data were available at the division level (the third administrative unit in Kenya at the time of design) from local Divisional Livestock Production Offices (DPLO) and, in the absence of any other information, were considered to be equally proportioned in each sub-location falling within each division’s boundary.

A number of random points, ranging between 1 and 8, were generated in each sub-location using ArcMap 9.2 (ESRI, Oakland, California) and the x and y co-ordinates entered into a Garmin eTrex hand-held geographical positioning system (GPS) via the DNR Garmin 5.4.1 extension for ArcView (Minnesota Department for Natural Resources, 2008). The GPS device was used to locate the physical location of each generated random point whilst in the field, and the nearest human habitation within 300 m was selected for recruitment. Distance between the point and habitations was assessed using the path distance function on the GPS. Where two or more habitations were within the same distance from the random point, the household that was closest to a north bearing was selected. In the absence of a household within 300 m, or following the household head’s refusal to participate, a ‘back-up’ point was randomly generated and recruitment followed in the same manner. Households were recruited regardless of livestock ownership status.

A household was defined as all people identified by the household head as being occupant at the time of recruitment and typically occupant (to the extent that food is regularly shared from the household pot(s)) within the past 4 weeks.

### Data collection within the household

On the sampling day, a detailed questionnaire was performed with the household head and covered data relating to household demography, access to services, known household level risk factors for infectious disease, and durable asset ownership. Each household occupant meeting the study inclusion criteria (5 years of age or older and not in the last trimester of pregnancy) was invited to participate and then interviewed on their education, occupation, food consumption history, contact with livestock and other animals, disease history and current state of health. When participants were less than 12 years of age, a guardian (preferably the mother) was asked to sit with them during questioning to assist with recall. Questionnaires were written in English and translated into the vernacular (Kiswahili, Dholuo or Kiluhya) during administration.

Following the questionnaire, a physical exam was performed on all participants by a study clinician (one of two medical officers) who also collected samples for diagnostic testing. A maximum of 25 ml of venous blood was collected from the forearm into plain, heparin, EDTA and Quantiferon-TB Gold (Cellestis Limited, Australia) tubes. Thin and thick blood smears were made using blood remaining in the butterfly apparatus following venous sampling. Participants were asked to provide a single faecal sample, collected from the first motion of the day into a collection pot left during recruitment.

At the same time, a veterinary team undertook a ~ 100 item questionnaire on livestock owned by the household with the main animal keeper. Individual level risk factor and other data were collected for all bovine and porcine animals within a household that met the inclusion criteria (3 months or older and not in the latter stages of pregnancy). We also recorded data from a physical examination of each animal, including age based on dentition, sex, temperature, anaemia as assessed by FAMACHA scoring [20] and body condition score [21]. In cattle, a total of 24 ml of blood was then collected through jugular venous puncture. Ear vein blood was collected using a sterile lancet and microhaematocrit tube from each animal, and used to prepare thick and thin blood smears. Faecal samples were collected per rectum from each animal. In pigs, 14mls of blood was sampled from the cranial vena cava. Ear vein blood was collected for preparation of thick and thin smears and per rectum faecal samples were collected where possible.

All data were recorded on a Personal Digital Assistant (PDA) data entry system (Aceeca MEZ1000 running the ‘Pendragon Forms’ software) and stored and managed in Microsoft Access databases. A barcode-based system was used to link biological samples to anonymised individuals and homesteads.

The geographic co-ordinates and altitude were collected at a central point within the homestead using the GPS.

### Laboratory processing: Human

Blood and faecal samples were tested for a wide range of infectious agents that were expected to be endemic in the study area.

Faecal samples were prepared using standard protocols for the Kato-Katz and Formal Ether techniques [22, 23] and examined under light microscopy. The presence or absence of helminth and protozoal gastrointestinal parasites was recorded and a quantitative estimate of the number of eggs per gram of faeces calculated where appropriate. Samples were additionally prepared using Ziehl-Nielsen staining to enable the identification of *Cryptosporidium* species [24]. Remaining material was stored in 5% saline with 0.3% Tween-20 at room temperature for subsequent analysis by copro-antigen ELISA for *Taenia* spp. [25].

Thick and thin blood smears were stained using 10% Giemsa and examined under 100× magnification with an oil immersion objective lens. Haemoparasites observed were recorded qualitatively (present/absent) and semi-quantitatively on the basis of standard intensity scales [24].

The buffy coat and the red blood cell/buffy coat interface from centrifuged haematocrit tubes containing heparinised blood were examined under 100× oil immersion and at the 10× power for the presence of *Trypanosomes* and *Ricketsiae* (the Haematocrit centrifugation technique, or the “Woo Method”) [26]. The buffy coat was transferred to a microscope slide and 100 fields examined at ×10 power for the presence of motile organisms.

Blood collected in serum tubes was spun at 3000 rpm for 20 min, and aliquoted into 2 ml barcoded cryovials before serological testing, or storage at −40 °C for later analysis. Serological assays included a rapid immuno-chromatographic flow assay (IgG, IgM) for exposure to *Brucella* spp. (Royal Tropical Institute, Netherlands); a commercial ELISA (IgG) (Serion-Virion GmbH, Germany) for *Coxiella burnetii* (Q-fever); an in-house indirect ELISA for Rift Valley fever (RVF) (IgG) [27] performed at Stanford University, USA; and a HP10-Ag ELISA for *Taenia solium* (cysticercosis) [28] supplied by Leslie Harrison, University of Edinburgh, UK. Heparinised human whole blood was tested for HIV infection using a rapid strip test (SD Bioline HIV 1/2 3.0) (Standard Diagnostics Inc., South Korea) and infection with *Mycobacterium* spp. was assessed using a gamma–interferon assay (QuantiFERON-TB test, Cellestis Limited, Australia).

### Laboratory processing: Animal

Blood and faecal samples were tested for a range of pathogens expected to be endemic in cattle and pigs in the study area including, where appropriate, the zoonotic pathogens tested for in people. A rapid immuno-chromatographic flow assay (IgG, IgM) was used for exposure to *Brucella* in cattle (Royal Tropical Institute, Netherlands). Cattle samples were tested for the presence of IgG antibodies to *Coxiella burnetii* using the Checkit Q fever ELISA (IDEXX). Rift Valley fever testing in cattle was performed using the ID Screen competitive ELISA (ID Vet, France). Cattle and pigs were tested for cysticercosis using the HP10-Ag ELISA. Heparinised blood from cattle was tested for *M. bovis* using the Bovigam® (Prionics, Switzerland) in vitro gamma-interferon assay. Faeces were processed using the McMaster technique, Baermans technique and qualitative sedimentation technique [29] as well as the Kato Katz technique [30] and examined by light microscopy. Blood smears were stained with Giemsa and examined for haemoparasites. The buffy coat and red blood cell/buffy coat interface was also examined for the presence of motile haemoparasites.

### Data analysis

#### Survey adjustment

We used design-based inference to adjust individual infection prevalence estimates and their standard errors on the basis of the complex study design in which individuals were nested in households and households nested in sublocations. Adjustment was implemented using the *svydesign* procedure in the *survey* package [31] in R statistical environment, version 3.1.1. (http://cran.r-project.org/). There were a large number of ‘singleton’ primary sampling units at the sublocation level (i.e. sublocations in which a single household was sampled), hence sublocations (*n* = 143) were aggregated by division (*n* = 17), which was used as a stratifying variable. A unique identifier was used for each household to account for clustering. Survey-adjusted prevalence and confidence intervals by age-group were derived for animals (cattle and pigs) and people.

Sampling weights were calculated as 1/*π*, where *π* is the sampling probability for each individual in each division, estimated as the fraction of the number of individuals sampled and the total number of people/animals per division. The total population size per division was derived from the 2009 census for people (https://opendata.go.ke/) and from DPLO data for cattle. In the absence of reliable pig population data at the division level, sampling weights were not included in the estimation of porcine prevalence data, which was only adjusted on the basis of household-level clustering.

The relationship between the prevalence of each human and animal infection and sex was assessed using the Wald statistic, as proposed by Koch et al. [32] for complex survey designs, and also implemented in the *survey* package in R.

#### Spatial analysis

Human and cattle exposure or infection risk was tested for evidence of spatial clustering using the spatial scan statistic [33] in SatScan version 9.0 (www.satscan.org). We used household-level infection (i.e. the presence or absence of at least one infected individual) as the outcome of interest. A Bernoulli model was used with 999 iterations (allowing estimation of *p*-values down to 0.001) and a cluster size up to a maximum of 20% of observations. Only those pathogens for which 10 or more households had at least one infected animal or human were included.

The spatial distribution of household livestock (cattle, pig, goat and sheep) ownership and household tribal affiliation was examined using a kernel smoothing approach [34]. For this, the kernel intensity of ‘positive’ households (e.g. households keeping cattle; membership of a particular tribal group etc) was divided by the kernel intensity of all sampled households in the study area. Hence, the numerator was number of ‘positive’ households per unit area for all parts of the study area while the denominator was the number of all households per unit area. The resulting ratio was considered to represent the probability of that outcome in a randomly selected household over the whole geographic dimensions of the study area. Household tribal affiliation was defined on the basis of the ethnicity reported by 50% or more of adults in each household, since household head ethnicity could not be ascertained from anonymised data. Kernel density surfaces were derived using the *sparr* package [35] in R, using a fixed bandwidth of 5 km and correction for edge effects.

## Results

A total of 416 households were recruited, with a total sample size of 2113 people. The average reported household size was 7.6 (range 1 to 30) people (including all age groups), from which our average household sample size was 5.1 (range 1 to 21). Of all eligible individuals present in households (2917), we were able to recruit 72.4%. Cattle were kept in 55.3% of households, from which we sampled 983 animals. The average herd size per household was 4.9, and we were able to sample 87.8% out of all animals in sampled households. Pig keeping was less common (16.9% of households), with an average herd size of 2.6. The total pig sample size was 91.

### Human infection

The survey adjusted individual human prevalence of infection with the range of pathogens under study is presented in Table 1. We did not observe (but considered possible) infection with *Isospora spp.*, *Cyclospora spp.*, *Dientamoeba fraglis*, *Trichostrongylus spp.*, *Schistosoma bovis* and *Wucheria bancroti.* Several infectious agents were highly prevalent, particularly hookworm (due to either *Ancylostoma duodenale* or *Necator americanus*) (36.3%), *Plasmodium falciparum* (the only malarial agent identified) (29.4%) and *Entamoeba histolytica/dispar* (30.1%)*.* Males were at significantly elevated risk of infection with *Strongyloides stercoralis*, *Schistosoma mansoni*, hookworm, and *P. falciparum.* Females were at significantly elevated risk of infection with *Taenia solium*, *Trichuris trichiura*, *E. histolytica/dispar* and HIV (Table 1).

**Table 1:** Survey adjusted individual and gender stratified prevalence estimates for the human infections under study

Infection	Adjusted prevalence (%, 95% CI)	Male (%)	Female (%)	*p* value^a^
Gastrointestinal parasites				
*Balantidium coli*	0.02 (0–0. 1)	-	-	-
*Fasciola* spp.	0.04 (0–0. 1)	-	-	-
*Entamoeba hartmanni*	0.1 (0–0.2)	-	-	-
*Endolimax nana*	0.1 (0–0.2)	-	-	-
*Hymenolepis* spp.	0.2 (0–0.3)	-	-	-
*Taenia* spp. (eggs)	0.3 (0–0.5)	-	-	-
*Blastocystis hominis*	0.6 (0.1–1.1)	-	-	-
*Cryptosporidium* spp.	0.6 (0.2–1.0)	-	-	-
*Strongyloides stercoralis*	2.9 (2.1–3.8)	3.9	2.1	0.023
*Giardia* spp.	3.2 (2.3–4.0)	4.0	2.5	0.09
*Taenia solium* (HP10-ELISA)	5.8 (4.4–7.2)	4.6	7.0	0.04
*Schistosoma mansoni*	5.9 (3.7–8.1)	7.2	4.8	0.009
*Trichuris trichiura*	10.0 (8.2–11.7)	7.6	12.0	0.002
*Ascaris lumbricoides*	10.4 (8.1–12.7)	9.7	11.1	0.33
*Iodamoeba butschlii*	14.2 (12.4–16.0)	13.4	15.0	0.42
*Taenia spp.* (Copro-ELISA)	19.7 (16.7–22.7)	20.7	18.8	0.29
*Entamoeba histolytica/dispar*	30.1 (27.5–32.8)	27.5	32.5	0.046
Hookworm	36.3 (32.8–39.9)	39.4	33.6	0.01
Haemoparasites				
*Plasmodium falciparum*	29.4 (26.8–32.0	32.1	27.0	0.02
Bacterial infections				
*Brucella* spp.	0.6 (0.2–0.9)	-	-	-
*Coxiella burnetii*	2.2 (1.5–2.9)	2.5	1.9	0.32
*Mycobacterium* spp.	8.2 (6.8–9.6)	7.8	8.5	0.64
*Viral infections*				
Rift Valley fever virus	0.5 (0.2–0.8)	-	-	-
HIV	5.3 (4.2–6.3)	2.9	7.3	<0.001

^a^Based on Wald Test with adjustment for survey design. Very rare outcomes not assessed

The risk of human exposure to zoonotic pathogens was relatively lower, with a very low prevalence of seropositivity to *Brucella* spp. (0.6%) and a moderately low prevalence of seropositivity to Rift Valley fever virus (1.5%) and *C. burnetii* (2.2%). No cases of human African trypanosomiasis (HAT) were identified using microscopy. A higher prevalence of *Mycobacterium* spp. (due to zoonotic or non-zoonotic species) was observed (8.2%). Zoonotic or potentially zoonotic protozoal agents, including *Cryptosporidium* spp. and *Balantidium coli* and zoonotic trematodes, such as *Fasciola* spp., were also rare (0.6, 0.02 and 0.04%, respectively). Infection with *Giardia* spp., which may be transmitted from livestock, was also found at moderately low prevalence (3.2%). Infection with *Taenia* spp. was more common, with a prevalence of 19.7% for taeniasis (the presence of either a *T. solium* or *T. saginata* worm in the gastrointestinal tract) based on a copro-antigen (Copro-Ag) test, and a prevalence of 5.8% for cysticercosis (the presence of circulating antigens from a *T. solium* cysticerci).

**Fig. 2:**
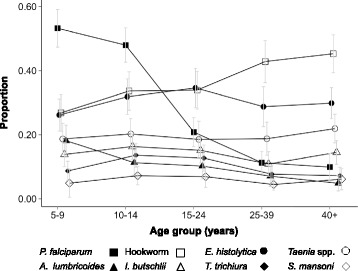
Age prevalence profiles for the common infections of people. Error bars represent 95% confidence intervals

There was evidence of some age structure to the prevalence of infection for the common pathogens of people (Fig. 2). This was most notable for *P. falciparum*, where the prevalence in the youngest age group was 53.2% (95% CI 47.3–59.1) compared to 9.9% (95% CI 5.7–14.1) in those people more than 40 years. Hookworm showed the reverse relationship, with a prevalence in children 5–9 years of 26.8% (95% CI 21.1–32.5), whilst this was 45.3% (95% CI 39.3–51.2) in people more than 40 years.

**Fig. 3:**
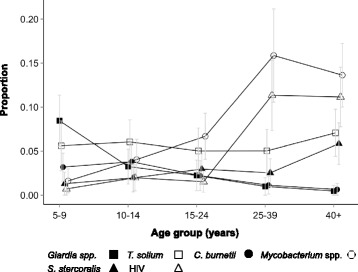
Age prevalence profiles for the rarer infections of people. Error bars represent 95% confidence intervals

HIV and TB infection showed similar age profiles to each other (Fig. 3), with risk being relatively low in children aged 5 to 9 (0.7% (95% CI 0.01–1.4) and 1.6% (95% CI 0.2–3.0), respectively), increasing to a peak in adults aged 25–39 (11.3% (95% CI 7.4–15.3) and 15.9% (95% CI 10.6–21.2)). Detectable antibodies for *C. burnetii* declined with age: children in the 5–9 age group had a prevalence of seropositivity of 3.2% (95% CI 1.6–4.8) whilst this was 0.6% (95% CI 0–1.3) in adults 40 years and above.

Out of a total of 22 possible infections (Table 2), and using combined data from Copro-ELISA and HP10-ELISA to represent all stages of *Taenia spp.* infection, 1544 individuals with complete data for all pathogens were infected with or had exposure to an average of 1.94 infectious agents, with a range of 0 to 6 and a median of 2. Two hundred and twenty two individuals (14.4%) had an infection/exposure count of zero while 196 (12.7%) had 4 or more, 57 (3.7%) 5 or more and 13 (0.8%) had 6 concurrent infections/exposures.

**Table 2:** Individual and gender stratified prevalence estimates for the cattle infections under study

Infection	Adjusted prevalence (%, 95% CI)	Male	Female	*p*-value^a^
Gastrointestinal parasites				
*Schistosoma bovis*	0.26 (0–0.55)	-	-	-
*Giardia* spp.	0.28 (0–0.66)	-	-	-
*Fasciola hepatica*	0.37 (0–0.94)	-	-	-
*Toxocara vitulorum*	0.98 (0.092–1.9)	-	-	-
*Nematodirus* spp.	1.4 (0.62–2.2)	-	-	-
*Dictyocaulus viviparus*	2.1 (1.2–3.0)	3.7	1.2	0.03
*Moniezia* spp.	3.1 (1.9–4.2)	3.0	3.1	0.96
*Strongoloides spp.*	4.0 (2.7–5.3)	5.9	3.0	0.07
*Trichuris* spp.	6.7 (4.8–8.6)	8.6	5.7	0.19
*Calicophoron daubneyi*	9.2 (6.7–11.5)	5.9	11.0	0.01
*Fasciola gigantica*	32.5 (27.6–37.3)	28.1	34.8	0.07
*Coccidia* spp.	37.2 (32.7–41.7)	44.3	33.3	0.002
*Strongyle* spp.	58.4 (53.8–63.0)	69.4	52.4	<0.001
Bacterial infections				
*Brucella* spp.	0.26 (0–0.56)	-	-	-
*Mycobacterium bovis*	2.2 (1.3–3.2)	3.3	1.7	0.16
*Coxiella burnetii*	10.0 (7.7–12.2)	8.2	10.9	0.20
Haemoparasites				
*Anaplasma* spp.	0.62 (0.05–0.28)	-	-	-
*Trypanosoma* spp.	5.8 (4.1–7.4)	4.3	6.6	0.12
*Theileria s*pp.	53.4 (48.6–58.3)	53.2	53.6	0.94
*Other parasites*				
*Taenia saginata*	53.5 (48.7–58.3)	53.7	53.2	0.92
*V* *iruses*				
Rift Valley fever virus	1.4 (0.55–2.22)	-	-	-

^a^Based on Wald Test with adjustment for survey design. Very rare outcomes not assessed

**Fig. 4:**
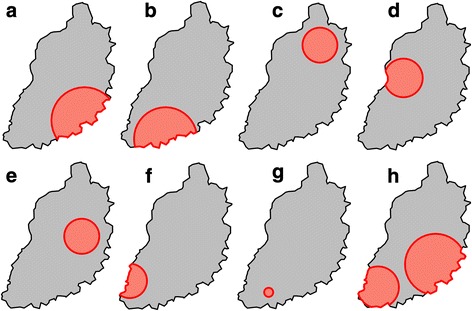
Clusters of elevated relative risk for household level infection with human pathogens: **a** *Ascaris lumbricoides*; **b** HIV; **c** Hookworm (*Ancylostoma duodenale* or *Necator americanus)*; **d** *Iodamoeba butschlii*; **e** *Plasmodium falciparum*; **f** *Schistosoma mansoni*; **g** *Taenia solium*; **h** *Trichuris trichiura*

Household infection with *A. lumbricoides*, *Brucella* spp. *Taenia* spp., *Cryptosporidium* spp., *C. burnetii*, *Entamoeba* spp., HIV, hookworm, *I. butschlii*, *P. falciparum*, *S. mansoni*, *S. stercoralis*, *Mycobacterium* spp., *T trichiura*, and *Giardia* spp. was examined for spatial clustering using the spatial scan statistic. Significant spatial clusters were detected for several of these infections (Fig. 4), specifically *T. trichiura* (Relative risk (RR) comparing households inside and outside identified cluster = 2.4, *p*-value = <0.001; RR = 2.1, *p*-value = 0.003), *A. lumbricoides* (RR = 2.4, *p*-value = 0.011), *Iodamoeba butschlii* (RR = 1.7, *p*-value = 0.004), HIV (RR = 2.6, *p*-value = 0.003), *S. mansoni* (RR = 5.7, *p*-value = <0.001), hookworm (RR = 1.4, *p*-value = 0.04), *T. solium* (RR = 5.3, *p* = 0.03) and *P. falciparum* (RR = 1.5, *p*-value = 0.002).

#### Cattle infection

The survey adjusted prevalence of individual animal infection is presented for cattle in Table 2. Over half of all animals were infected with *Strongyle* spp. (58.4%) and *Theileria* spp. (53.4%). Infection with *Coccidia* spp. (37.2%) and *Fasciola gigantica* (32.5%) was also very common. Male animals were more likely to be infected with *Coccidia* and *Strongyle* spp., while females were more likely to be infected with *Calicophoron daubneyi*. There was weaker evidence for an effect of sex on *F. gigantica*. Although infection with *Dictyocaulus viviparous* (lungworm) was relatively rare (2.1%), there was some evidence that males were at elevated risk (Table 2).

The prevalence of seropositivity to *Brucella* species was very low (0.26%). The prevalence of seropositivity to Rift Valley fever virus and *Mycobacterium bovis* were both moderately low (1.4 and 2.2%, respectively). Ten per cent of animals were seropositive for *Coxiella burnetii* while over 50% of animals showed evidence of cysticercosis due to *T saginata*. *Trypanosoma* spp. infections were found in nearly 6% of animals. Of these 73.2% were considered to be non-zoonotic *T. vivax*, 12.5% *T. theileri* and 14.3% *T. congo* based on morphology.

**Fig. 5:**
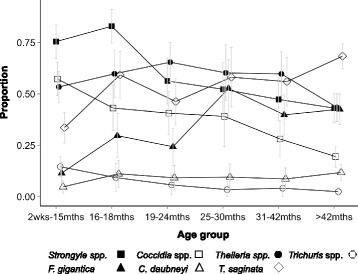
Age prevalence profiles for the common infections of cattle. Error bars represent 95% confidence intervals

**Fig. 6:**
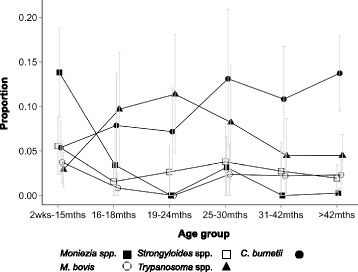
Age prevalence profiles for the rarer infections of cattle: Error bars represent 95% confidence intervals

Infections with *Strongyle*, *Coccidia* and *Trichuris* spp. were most common in younger animals (75.5% (67.3–83.6), 57.3% (49.2–65.3), 14.6% (9.8–19.3) in animals less than 15 months, compared to 43.1% (36.1–50.1), 19.5% (14.4–24.5), 2.4% (0.4–4.4) in animals more than 42 months, respectively), whilst a reverse trend was seen for *F. gigantica* (11.5% (6.4–16.6) versus 42.6% (35.1–50.0)) (Fig. 5). Detectable antibodies to *C. burnetii* increased with animal age, with animals aged less than 15 months having a prevalence of 5.4% (2.4–8.4) compared to 13.7% (9.5–18.0) in animals aged more than 42 months. This was also the case for *T. saginata*, where the youngest age group had a prevalence of 33.6% (26.1–41.1) compared to 68.4% (62.4–74.3) in the oldest (Fig. 6).

Out of the 21 pathogens listed in Table 2, the 601 cattle with complete data for all pathogens were infected with (or had exposure to) an average of 2.8 pathogens, with a range of 0 to 7 and median of 3. Most animals had at least one exposure/infection (97.3%), with 84.2% having 2 or more, 57.9% 3 or more, 28.0% 4 or more, 10.1% 5 or more, 2.3% with 6 or more and 0.3% (2 animals) with 7 exposures/infections.

**Fig. 7:**
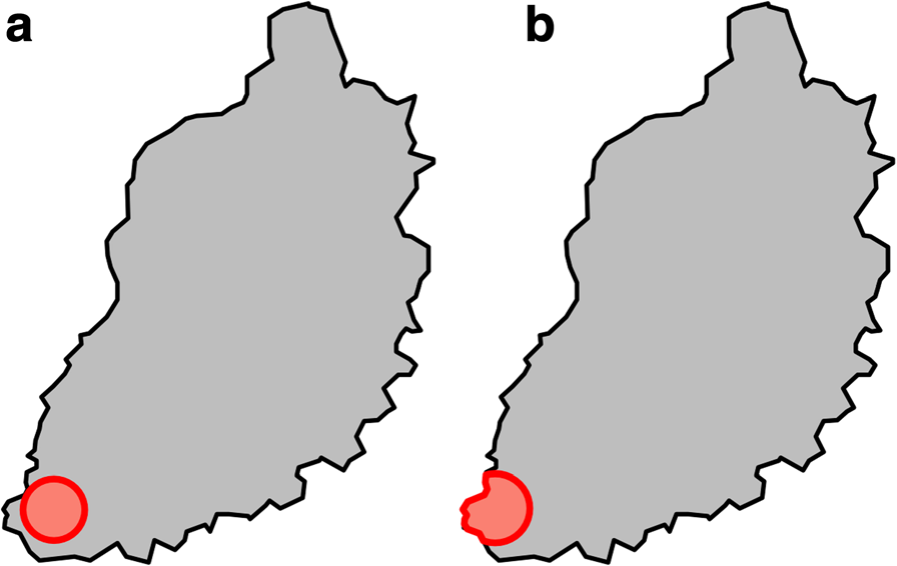
Clusters of elevated relative risk for household level infection with cattle pathogens: **a** *Calicophoron daubneyi*; **b** *Trypanosoma* spp.

Household-level animal infection with *Theileria parva*, *Trypanosome* spp., *C. daubneyi*, *F. gigantica*, *Coccidia* spp., *Trichuris* spp., *Nematodirus* spp., *Moniezia* spp., *Strongyle* spp., *S. papillosus, Mycobacterium bovis, T. saginata* and *C. burnetii* was examined for spatial clustering using the spatial scan statistic. Only *C. daubneyi* (RR = 3.8, *p*-value = 0.03) and *Trypansoma* spp. (RR = 5.8, *p*-value = 0.01) showed significant spatial clustering. Both clusters overlapped to a large extent, and were found on the border of Lake Victoria in the south west of the study area (Fig. 7).

#### Pig infection

The limited number of pigs sampled were heavily parasitised, with almost all (91.7%) having *Strongyle* infections and around half having *Strongyloides*, *Coccidia* or *Ascaris* spp. infection (Table 3). 17.2% of animals also appeared to be infected with potentially zoonotic *T. solium* cysts. There was no evidence of differences between the sexes in risk for any infection.

**Table 3:** Individual and gender stratified prevalence estimates for the pig infections under study

Infection	Adjusted prevalence (%, 95% CI)	Male	Female	*p*-value^a^
*Babesia* spp.	1.2 (0–3.4)	-	-	-
*Theileria* spp.	1.1 (0–3.4)	-	-	-
*Trypanosome* spp.	3.2 (0–9.3)	-	-	-
*Taenia solium*.	17.2 (9.1–25.3)	14.6	19.2	0.62
*Trichuris* spp.	25.0 (13.7–36.3)	26.9	23.5	0.74
*Ascaris* spp.	46.7 (33.7–59.6)	38.5	52.9	0.29
*Strongyloides* spp.	50.0 (34.7–65.3)	50	50	1
*Coccidia* spp.	55.0 (40.4–69.6)	61.5	50	0.36
*Strongyle* spp.	91.7 (83.3–1)	88.5	94.1	0.38

^a^Based on Wald Test with adjustment for survey design. Very rare outcomes not assessed

Out of the 9 infectious agents listed in Table 3, pigs had an average of 2.9 unique infections, with a range of 1 to 6 and a median of 3. All pigs had at least one infection.

#### Study area demography

**Fig. 8:**
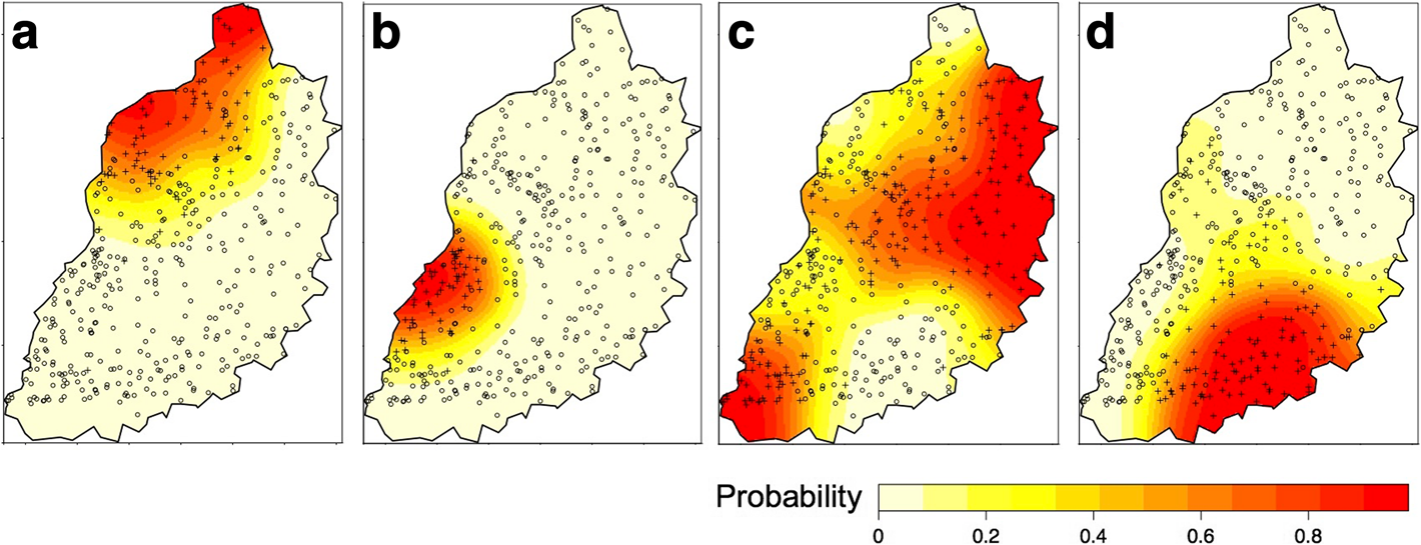
Probability that household belongs to tribal group (**a** Teso; **b** Samia; **c** Luhya; **d** Luo)

The majority (69.4%) of study participants reported attaining at least primary level education. The main tribe was Luhya (50.2%) followed by Luo (21.9%), Teso (14.5%) and Samia (12.6%), with a small number of participants (0.7%) belonging to the Kikuyu, Saboat, Turkana, Kuria, Kalenjin, Pokot or Muganda tribes. The main tribal groups were highly spatially aggregated (Fig. 8). The majority (96%) of participants were Christians (of Roman Catholic, Pentacostal, Protestant or Baptist denominations) with 1.9% of participants being Muslim, and less than 1% of participants reporting to practise a tribal religion or to belong to no religion.

#### Livestock keeping

**Fig. 9:**
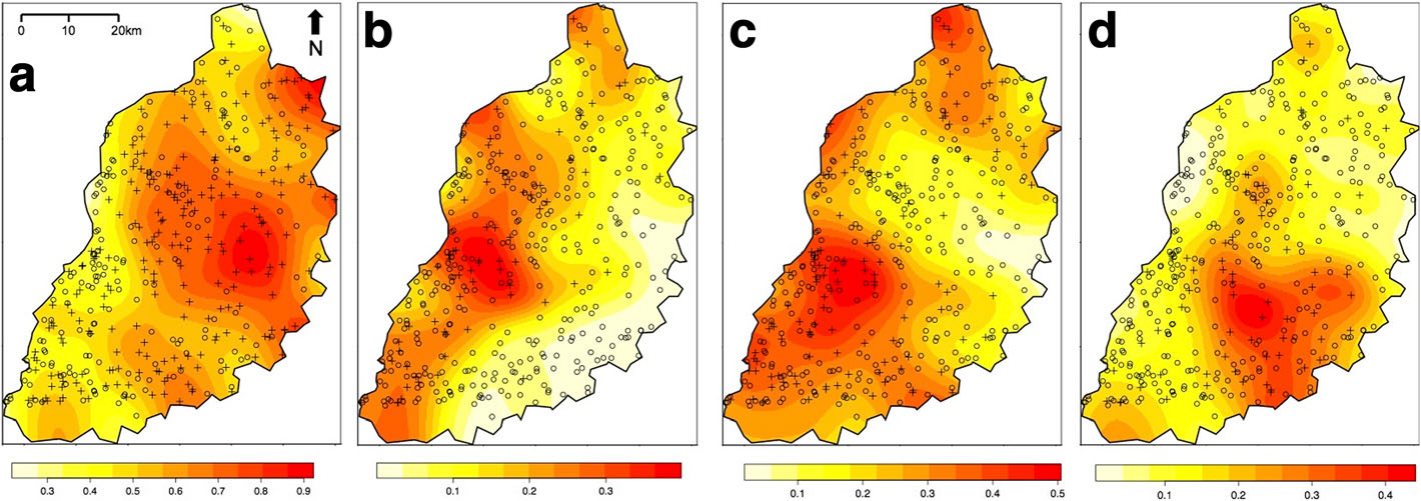
Probability that household owns livestock species (**a** Cattle; **b** Pigs; **c** Goats; **d** Sheep)

Most households kept livestock (92.5%), with the most common species being chickens (87.2%). Goats were kept in 27.4% of households, sheep in 15.6% and ducks in 11.1%. Cats and dogs were also kept by many of the households (48.9 and 35.9%, respectively). The spatial distribution of large and small ruminant and pig keeping households is shown in Fig. 9 and suggests some spatial structuring, particularly for pigs, sheep and goats.

In addition to high rates of household livestock ownership, potentially important exposures for zoonotic transmission were common in this community. Almost half of the study participants reported taking animals out for grazing at least weekly (47.2%); 14.2% reported milking cattle at least weekly; 6.7% of households reported having direct involvement in animal parturition in the past year; 15% reported having direct involvement in the slaughter of animals in the past year; and 5% reported hunting in the past year. Most individuals (85.3%) reported regularly seeing rats around the household.

#### Food preferences

Meat consumption was common in the community, with 86.2% of participants reporting eating meat (65.5% pork, 84.6% beef). Reported sources included butcheries (89.6%), neighbours (13.4%), and more rarely from own animals (3.2%). Approximately 75% of respondents ate meat outside the home. Most study participants reported consuming cows milk (95%), with 87.4% reporting that they boil it before consumption. 4% of participants reported consuming goats milk. Animal blood was consumed by 20% of participants.

#### Awareness of Zoonoses

Only 15.1% of respondents were aware that infectious diseases can be acquired from animals. Of these, 5.6% named anthrax as a zoonotic disease that could be acquired from cattle; 22.9% named brucellosis from cattle, but only 8.3% from sheep and goats; 12.3% reported that cattle were involved in sleeping sickness transmission; 10.6% that TB could be acquired from cattle; 18.3% that tapeworm could be acquired from meat; and 33.1% that rabies was transmitted from dogs.

## Discussion

The “People, Animals and their Zoonoses” study uses a novel human and animal co-sampling approach, which moves away from the ‘one-host, one-pathogen, one-outcome’ paradigm. We report here on the prevalence of infection with a very wide range of pathogens of both people and livestock within a single community. We intend that this integrated survey of human and animal health will allow the development of evidence-based recommendations for the control of zoonotic and other diseases within this mixed farming area of Kenya. The methodology applied is one that can be repeated elsewhere in different communities and environments. Indeed, doing so would provide valuable multi-site data for comparative analysis.

A number of infectious agents were highly prevalent within this community, and polyparasitism is common. We therefore repeat the findings from studies from a number of other communities in low income settings [36–38], and provide further evidence of the utility of considering multiple pathogens within single systems. Significant co-infections between particular pathogens may suggest commonalities in exposure, which may provide targets for integrated control [39, 40]. Alternatively, it may point to important biological interactions in the establishment, replication, and persistence of infection [41]. The wide range of pathogens considered here provides a rich data set for exploration of such relationships. In particular, by considering infection with the ‘big 3’ infectious diseases (HIV, TB and malaria), in addition to a number of neglected zoonotic and tropical diseases, these data can potentially contribute to the growing evidence base on the effects of immunological interactions between these pathogens on within community transmission dynamics [18, 42, 43].

We show that infection risk is not homogeneous across the study area, and that spatial heterogeneities in the probability of household infection exist for several of the human and animal pathogens studied. Exploratory spatial analysis can provide a powerful means with which to identify spatially heterogeneous contextual effects. Such effects might explain why disease risk varies in individuals with the same individual characteristics, but living in different social or biophysical environments [44, 45]. Alternatively, clustering of adverse health outcomes may occur as a result of compositional effects, or aggregations of individual risk factors within certain regions [46]. Future work will involve analytical studies that seek to disentangle some of these contextual and compositional effects on individual infection risk [47]. Given the substantial spatial structuring of household ethnicity, it seems likely that tribe may be an important compositional effect for several human infectious diseases in the study area.

Despite widespread livestock ownership, and regular reported contact with livestock, the prevalence of brucellosis, Q-fever and Rift Valley fever were all low in people. The prevalence of brucellosis was also extremely low in cattle. Bovine brucellosis is known to occur in Kenya, but is likely to have a highly heterogeneous distribution: a seroprevalence survey of cattle in three areas of Kenya revealed highest prevalence in pastoral areas of Samburu (15%), lowest in a tropical highland climate in Kiambu district (2%), adjacent to Nairobi in the central highlands of Kenya and intermediate prevalence in Kilifi district (10%), a lowland area on Kenya’s coast [48]. Rates of human brucellosis are also likely to be highest in pastoral areas of the country [49, 50]. It may be the case that herd sizes are currently too small, or there is insufficient mixing between herds to facilitate *Brucella* spp. transmission within this small holder farming community.

Much less work has been done on Q-fever in Kenya than on brucellosis, although a recent study indicated it was an important, but typically undiagnosed, cause of febrile illness in western Kenya [51]. The prevalence of exposure was considerably higher than to *Brucella* spp. in cattle, and further work to explore the importance of cattle ownership and contact on human risk of infection will be enlightening. RVF virus has not previously been reported in western Kenya, although epidemics have occurred in neighboring regions [52]. Further work is underway to examine whether the low prevalence observed in this study suggests inter-epidemic transmission occurs in western Kenya, including a cross-sectional survey of RVF prevalence in high risk slaughterhouse workers in the same area [53].

*Taenia spp.* are highly prevalent in the study area, with the high levels of human taeniasis and bovine and porcine cysticercosis observed being an important public health concern [54]. It should be noted that the HP10 Antigen ELISA cross-reacts with *Taenia hydategena* [55] which may lead to an over-estimation of *T. solium* prevalence in the pig population. The prevalence of *T. hydategena* in African pigs has historically been presumed to be low, although a recent study in Tanzania suggested a prevalence of 6.6% [56]. The prevalence of human taeniasis in this community is nearly 20%. A similarly high prevalence of almost 30% was previously identified in hyper-endemic foci in South-East Asia [57]. The copro-Ag ELISA for the identification of human taeniasis is not species-specific, detecting both *Taenia saginata* and *solium* [58]. Household-level human taeniasis was found to be spatially clustered in the study area, and whilst we did not find evidence of clustering in household-level bovine cysticercosis, further work is underway to explore the spatial distribution of human taeniasis and bovine and porcine cysticercosis at the individual and household level (http://journals.plos.org/plosntds/article?id=10.1371/journal.pntd.0004223). Three adult *Taenia spp*. worms collected from individuals found to be tapeworm carriers based on microscopy in this study were identified as *T. saginata* by PCR at the Institute for Tropical Medicine in Antwerp, Belgium. An additional important output of this work is the identification of widespread lack of awareness of zoonotic disease; a series of health education messages about these diseases, particularly the risks associated with *Taenia* spp. and messages around food safety are likely to be valuable.

Kenya is undergoing rapid changes in livestock production in order to meet the demands of a growing, increasingly urban, population. This is leading to a trend towards the intensification of livestock production and wider marketisation of livestock products in many parts of the country, including in western Kenya [59]. This comprehensive study provides a baseline for the prevalence of zoonotic infection in both people and animals in a farming community that can contribute to the monitoring of how changing agricultural systems may impact on the dynamics of zoonotic disease transmission.

## Conclusion

This large, multi-disciplinary study provides a comprehensive overview of the prevalence of a wide range of pathogens of people and animals in a smallholder farming community in rural western Kenya. This integrated study fits very much within the one health paradigm, and will allow a range of hypotheses about human and animal disease in these linked populations to be tested. A major aim of future work will be to explore the determinants of individual and household infection with single and multiple pathogens in the context of a range of social, environmental and physical parameters. Our rich dataset will also allow exploration of conditions such as polyparasitism and parasite co-occurrence, and in particular how zoonotic pathogens fit into the broader ecology of endemic infectious disease in the study area.

## Abbreviations

CI: Confidence interval
Copro-Ag: Copro-antigen
DPLO: Divisional livestock production office
ECF: East Coast fever
ELISA: Enzyme-linked immunosorbant assay
GPS: Geographical positioning system
HAT: Human African trypanosomiasis
HIV: Human immunodeficiency virus
IgG: Immunoglobulin G
IgM: Immunoglobulin M
NZD: Neglected zoonotic disease
PAZ: the “People, Animals and their Zoonoses” study
PCR: Polymerase chain reaction
RR: Relative risk
RVF: Rift Valley fever
TB: Tuberculosis
